# Effects of Stocking Density on Growth Performance, Physiological Responses, and Transcriptomic Profile of *Penaeus monodon*

**DOI:** 10.3390/ijms27031505

**Published:** 2026-02-03

**Authors:** Jianzhi Shi, Wenzhe Li, Song Jiang, Hongshan Diao, Jianhua Huang, Lishi Yang, Yundong Li, Yangyang Ding, Yafei Duan, Xu Chen, Qibin Yang, Falin Zhou

**Affiliations:** 1 Key Laboratory of Efficient Utilization and Processing of Marine Fishery Resources of Hainan Province, Sanya Tropical Fisheries Research Institute, Sanya 572018, China; shijianzhi1989@163.com (J.S.); chenxu1@scsfri.ac.cn (X.C.); 2 Key Laboratory of South China Sea Fishery Resources Exploitation and Utilization, Ministry of Agriculture and Rural Affairs, South China Sea Fisheries Research Institute, Chinese Academy of Fishery Sciences, Guangzhou 510300, China; m15836677553@163.com (W.L.); tojiangsong@163.com (S.J.); 17875808801@163.com (H.D.); huangjianhua@scsfri.ac.cn (J.H.); yangls2016@163.com (L.Y.); liyd2019@163.com (Y.L.); dingyangyang93@163.com (Y.D.); duanyafei@scsfri.ac.cn (Y.D.); 3 State Key Laboratory of Biocontrol/Southern Marine Science and Engineering Guangdong Laboratory (Zhuhai), School of Life Sciences, School of Marine Sciences, Sun Yat-sen University, Guangzhou 510275, China

**Keywords:** *Penaeus monodon*, stocking density, growth performance, WGCNA, oxidative stress, immune, homeostasis

## Abstract

Stocking density plays an important role in *P. monodon* aquaculture. However, its underlying molecular mechanisms remain unknown. In this work, after a 40-day culture period, we studied the effects of different stocking densities (G1: 50, G2: 100, G3: 150 ind/m^3^) on *P. monodon*, assessing their growth performances, physiological indexes, and transcriptome profiles. It was found that increased stocking density greatly decreased the growth performance and survival rate as well as the level of immune enzymes (alkaline phosphatase, acid phosphatase) and antioxidant component (total antioxidant capacity, superoxide dismutase, reduced glutathione) activities. Transcriptomic analysis showed that there are 2284 differentially expressed genes in all groups. Enrichment analysis of WGCNA results indicated that the green module associated with G2 was enriched for amino acid metabolism, lipid metabolism and immunity-related pathway. Furthermore, the G3 associated turquoise module was dominated by stress response, detoxification and energy metabolism pathways. Together, high stocking density causes the occurrence of oxidative stress, disturbance to immune system and alteration of metabolism profiles in *P. monodon*, whereas medium density (G2) is favorable to maintain physiological homeostasis. The results provide theoretical support to optimize aquaculture practice and contribute valuable information for subsequent studies.

## 1. Introduction

Aquaculture is among the fastest growing sectors in world agriculture. Currently, it supplies most of the fish and shellfish to meet the demand from humans more than the capture fisheries in last few decades [1]. Among the aquaculture species, shrimp aquaculture is particularly important to meet increasing demands for animal protein. The production of shrimp grew rapidly over the last few years, with species like *Litopenaeus vannamei* and *Penaeus monodon* being predominant in the industry [2]. It is worth mentioning that *P. monodon* is an important species cultured in several tropical and subtropical countries due to its high market value and ability to thrive under different conditions [3]. In 2023, the production of *P. monodon* cultured in China reached 128,000 tons [4]. However, challenges to productivity and sustainability persist even at high levels of intensification.

To maximize yield and profitability, shrimp farming models have changed gradually, from low stocking densities and extensive pond systems to high-density, controlled-environment systems [5]. In China, for example, the aquaculture of *P. monodon* has evolved from traditional earthen pond to industrialized recirculating systems. A key feature of intensive shrimp farming is high stocking density. It has been reported that high stocking density could lead to compromised water quality, increased disease susceptibility, and chronic stress [6,7]. These factors impair the growth performance and survival rates of shrimp, finally resulting in economic losses [8,9]. Consequently, intensified management is required for high-density culture of *P. monodon*.

In aquaculture, stocking density is considered as an important rearing factor affecting production costs, behavioral interactions, and physiological reactions [10]. Various studies in crustaceans indicate that high density is associated with decreased growth, altered feeding efficiency, and suppressed immune function [11,12,13]. In *Fenneropenaeus chinensis*, high densities have been shown to negatively impact growth performance by influencing the activities of enzymes such as phenol oxidase (PO) and peroxidase (POD) [14]. A similar trend was also found in *L. vannamei*, where high density was shown to exacerbate oxidative stress and lower resistance to pathogens such as *Vibrio* spp. [15]. Limited research is available on the effects of stocking density on *P. monodon*. Existing studies have mainly focused on growth and mortality parameters, with limited exploration of molecular response to different stocking densities [16,17].

The recent development of transcriptomics technologies allows for a more detailed investigation into the response of aquatic animals to environmental stresses by examining their gene expression profiles [18]. To date, transcriptome sequencing has been applied to explore immune and metabolism-related genes in *L. vannamei* and *Marsupenaeus japonicus*, which provides valuable information on stress adaptation [19,20]. In terms of *P. monodon*, previous studies based on transcriptomic analysis were focused on environmental stresses, such as changes in salinity or temperature, which revealed crucial stress-response genes and pathways [21,22]. However, information about how *P. monodon* transcriptionally responds to high-density culture conditions remain limited, which has blocked the progress of the breeding and aquaculture industries.

This research aims to fill the information gap by assessing the effects of varying stocking densities on *P. monodon*. Specifically, we evaluated the impacts on growth performance, key enzyme activities and transcriptomic regulation. By integrating physiological measurement with high-throughput sequencing, we seek to gain a comprehensive understanding of the molecular mechanism linked to stocking density. Our findings will provide a theoretical basis for optimal stocking density and guide the culture management of *P. monodon*.

## 2. Results

### 2.1. Growth Performance Metrics

**Figure 1:**
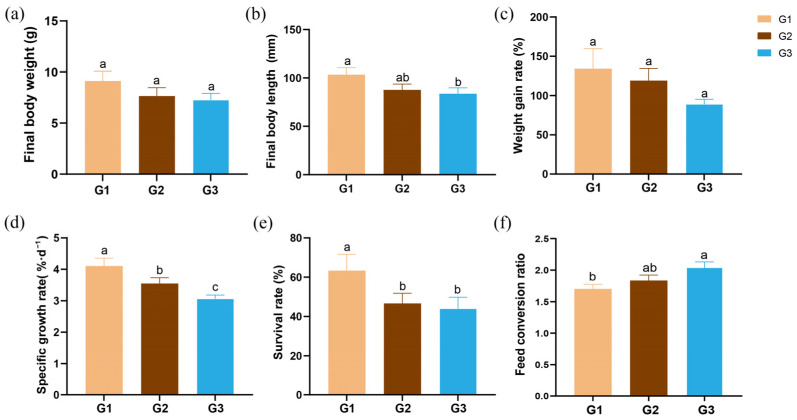
Effects of stocking density on the growth performance of *P. monodon*. (**a**) Final body weight; (**b**) Final body length; (**c**) Weight gain rate; (**d**) Specific growth rate; (**e**) Survival rate; (**f**) Feed conversion ratio. Different superscript letters indicated significant differences exist among treatments (*p* < 0.05).

The growth performance metrics of *P. monodon* are shown in Figure 1. As stocking density increased, a decline was observed in final body weight (FBW), final body length (FBL), weight gain rate (WGR), specific growth rate (SGR) and survival rate (SR). Significant differences in SGR were detected across all three experimental groups (*p* < 0.05). Specifically, shrimps in the high stocking density (G3) group exhibited significantly lower FBL and SR compared to those in the low stocking density (G1) group (*p* < 0.05). In terms of feed conversion ratio (FCR), it was significantly higher in the G3 group than in the G1 group.

### 2.2. Immunity and Antioxidant Activities in Hepatopancreas

**Figure 2:**
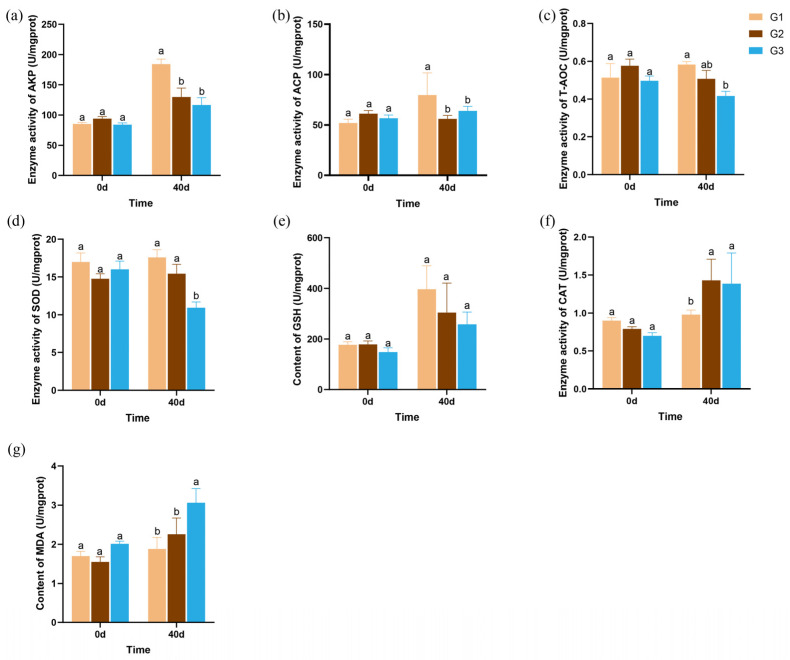
Effects of stocking density on the immunity and antioxidant activities in hepatopancreas after 0 days and 40 days of cultivation. (**a**) Alkaline phosphatase; (**b**) Acid phosphatase; (**c**) Total antioxidant capacity; (**d**) Superoxide dismutase; (**e**) Reduced glutathione; (**f**) Catalase; (**g**) Malondialdehyde. Different superscript letters indicated significant differences exist among treatments (*p* < 0.05).

The immunity and antioxidant activities were measured at two time points. The 0 d (onset of the experiment) samples served as the baseline for assessing the initial physiological state, while the 40 d samples were used to evaluate the effects of stocking density. As shown in Figure 2, the immune and antioxidant parameters showed no significant differences among the three groups on day 0. After 40 days of cultivation, significant decreases in alkaline phosphatase (AKP) and acid phosphatase (ACP) activity were observed in the G2 and G3 groups compared with the G1 group (Figure 2a,b). Key components of the cellular antioxidant system, such as total antioxidant capacity (T-AOC), superoxide dismutase (SOD) were significantly reduced in the G3 group (*p* < 0.05; Figure 2c,d). No significant differences were observed in reduced glutathione (GSH) content among the groups (Figure 2e). However, the activity of catalase (CAT) significantly increased under high stocking density conditions, suggesting a compensatory role in scavenging reactive oxygen species (*p* < 0.05; Figure 2f). In addition, the content of malondialdehyde (MDA) increased significantly in the G3 group (*p* < 0.05; Figure 2g), suggesting elevated oxidative damage at higher stocking density.

### 2.3. Summary of mRNA Sequencing Data

To investigate the effects of stocking density on mRNA expression, nine RNA libraries were constructed from shrimp hepatopancreas and subjected to high-throughput sequencing. As summarized in Table 1, a total of 442,408,418 raw reads were obtained. Following stringent quality filtering, 442,393,032 clean reads were retained across all libraries, representing 99.99% of the raw data.

### 2.4. Assessment of Relationships Among Samples and Repeatability

**Figure 3:**
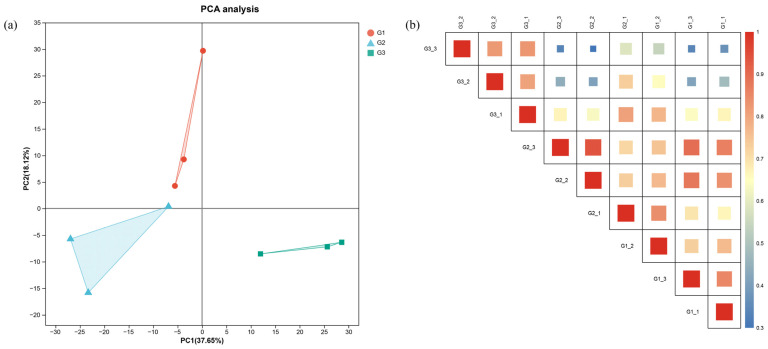
Relationships among samples. (**a**) PCA analysis; (**b**) Pearson correlation analysis.

To assess the relationships between samples, principal component analysis (PCA) was performed based on the mRNA expression data (Figure 3a). The results showed that the three replicates of each group form a relatively compact cluster. Similarly, the Pearson correlation analysis also indicated high intra-group sample correlations and clustering (Figure 3b). Furthermore, segregation was observed among the groups, suggesting that the expression patterns of genes were substantially altered with stocking densities.

### 2.5. Identification of Differentially Expressed Genes

**Figure 4:**
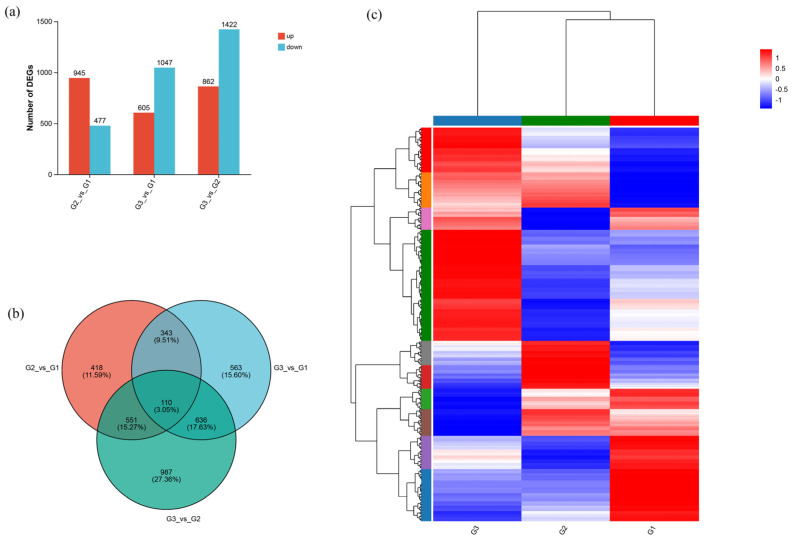
Identification and comparative analysis of DEGs. (**a**) Number of DEGs; (**b**) Venn diagram of DEGs; (**c**) Heatmap analysis of DEGs.

Pairwise comparisons were performed to identify differentially expressed genes (DEGs). Compared to the G1 group, the G2 group exhibited 1422 DEGs, of which 945 were up-regulated and 477 were down-regulated. More DEGs were observed between the G1 and G3 groups. A total of 1652 genes were analyzed, of which 605 were up-regulated and were 1047 down-regulated were identified. The largest difference in gene expression was found between the G2 and G3 groups, which contained 2284 DEGs (862 up-regulated and 1422 down-regulated) (Figure 4a).

A Venn diagram of the DEGs revealed distinct gene expression profiles across the comparison groups. We found that 110 genes were differentially expressed across all stocking density conditions (Figure 4b). Based on the heatmap analysis, distinct clustering patterns of DEGs were clearly observed among the three experimental groups (Figure 4c).

### 2.6. Weighted Gene Co-Expression Network Analysis (WGCNA) and Key Module Screening

**Figure 5:**
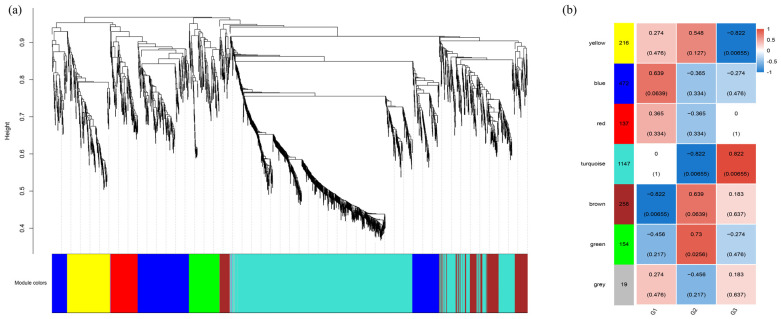
Weighted gene co-expression network analysis (WGCNA) of all DEGs. (**a**) Hierarchical cluster tree (cluster dendrogram) constructed with WGCNA; (**b**) Heatmap of relationships between modules and groups.

Gene co-expression networks were generated by calculating pairwise correlation coefficients of expression levels across all samples. A dendrogram was constructed from all DEGs of the nine libraries (Figure 5a). The primary branches of this clustering tree were then designated as distinct modules. Seven functional modules were identified, which contained a variable number of genes, spanning from 19 to 1147. Then, a heatmap was constructed to display the correlation matrix between the identified modules and the experimental groups (Figure 5b). The analysis revealed that the green and turquoise modules exhibited significant positive correlations with the G2 and G3 groups, respectively (*p* < 0.05). Although a strong correlation coefficient was observed between the blue module and the G1 group, this association was not statistically significant (*p* > 0.05). As a result, the green and turquoise modules were selected and submitted to further functional enrichment analysis.

### 2.7. Functional Enrichment Analysis of Key Modules

**Figure 6:**
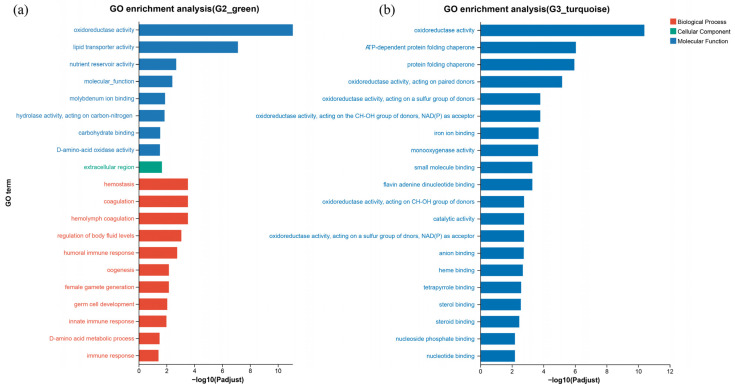
GO enrichment analysis of DEGs. (**a**) Significantly enriched GO terms in green module related to G2; (**b**) Significantly enriched GO terms in turquoise module related to G3.

To gain insight into the dynamic molecular response at different stocking densities, GO enrichment analyses of DEGs grouped in key modules were performed (Figure 6). The functional landscape of the green module (G2 group associated) was characterized by a combination of immune defense, reproductive development, and basic metabolism. Key enriched terms included immune responses, oxidoreductase activity, lipid transport, and nutrient reservoir activity (Figure 6a). In contrast, the turquoise module (G3 group associated) exhibited a highly specialized functional signature dominated by metabolic catalysis and molecular binding. The most significant pathway enriched was multiple oxidoreductase activity and related GO terms including monooxygenase activity, iron ion binding and flavin adenine dinucleotide. Chaperone functions in protein folding were also enriched (Figure 6b).

**Figure 7:**
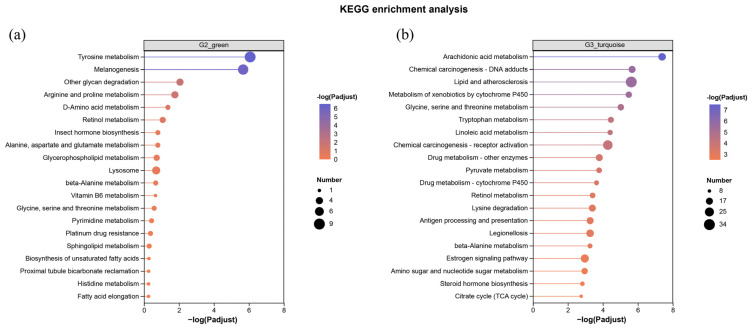
KEGG enrichment analysis of DEGs. (**a**) Significantly enriched pathways in green module related to G2; (**b**) Significantly enriched pathways in turquoise module related to G3.

Distinct metabolic and physiological profiles of key modules were revealed by KEGG pathway enrichment analysis. In the green module, a large number of pathways related to basal metabolism was enriched. These notably included multiple amino acid metabolic pathways such as tyrosine, arginine and proline, and glycine, serine and threonine metabolism. In addition, pathways involved in lipid metabolism (e.g., glycerophospholipid and sphingolipid metabolism, biosynthesis of unsaturated fatty acids, fatty acid elongation) were also significantly represented (Figure 7a). For the turquoise module, the enriched pathways indicated a pronounced shift toward disease and defense responses. Key pathways encompassed lipid-mediated inflammatory processes (e.g., arachidonic acid and linoleic acid metabolism, lipid and atherosclerosis) and multiple pathways associated with chemical carcinogenesis and general pathways in cancer. Metabolism of xenobiotics by cytochrome P450 and other drug-metabolizing enzymes remained prominent. Furthermore, pathways related to energy metabolism (pyruvate metabolism, citrate cycle, lysine degradation), pathogens and immune response (legionellosis, antigen processing and presentation) were also enriched in this group (Figure 7b).

### 2.8. Quantitative Real-Time PCR Validation of DEGs

**Figure 8:**
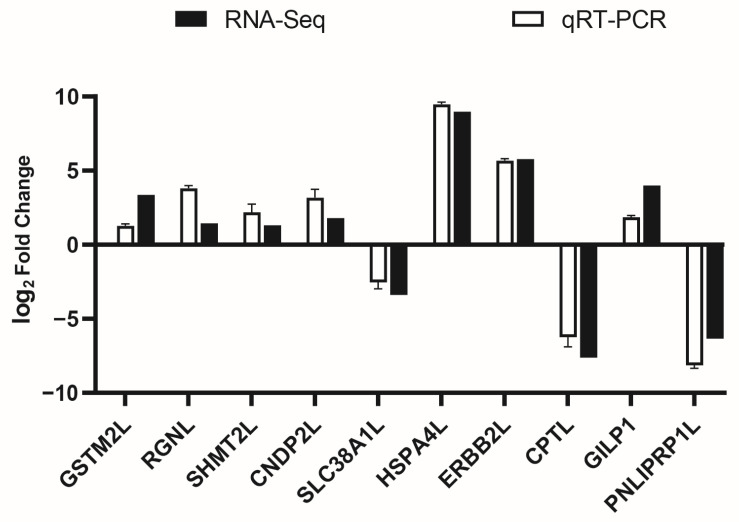
Validation of DEGs by qPCR assay.

To validate the accuracy of mRNA-seq data, ten random DEGs were selected for qPCR analysis. These comprised seven up-regulated genes (*GSTM2L*, *RGNL*, *SHMT2L*, *CNDP2L*, *HSPA4L*, *ERBB2L*, and *GILP1*) and three down-regulated genes (*SLC38A1L*, *CPTL*, and *PNLIPRP1L*). The expression trends determined by qPCR were consistent with the sequencing data (Figure 8).

## 3. Discussion

Stocking density is considered as a chronic stressor which could impact the growth of aquatic species. In crustaceans, several reports have shown that higher stocking densities generally result in lower growth metrics, such as weight gain and specific growth rate [5,23,24]. In accordance with this established consensus, the present study demonstrated a clear negative effect of increasing stocking density on the growth performance of *P*. *monodon*. Increasing density (from G1 to G3) led to a progressive decline in FBW, WGR, SGR, and SR. Importantly, SGR showed significant differences across all density groups, and survival in the highest density group (G3) was significantly lower than in the lowest (G1). These results agree with earlier findings that high-density culture induces physiological stress sufficient to impair both growth efficiency and survival in penaeid shrimp [25,26,27], confirming that growth performance of *P. monodon* is inversely related to stocking density.

In invertebrates such as shrimp, which lack an acquired immune system, defense against pathogens relies entirely on non-specific immunity [28]. Key enzymes involved in this immune response, including ACP and AKP, are widely used as markers for measuring the level of immunity [29]. In this study, the activities of ACP and AKP were measured to evaluate the non-specific immune capacity of *P. monodon* at various stocking densities. The results demonstrated a significant reduction in the ACP and AKP activity at higher stocking densities. A similar trend was observed in *Palaemonetes sinensis*, where ACP activity in groups at 5, 10, and 20 individuals/L was significantly lower than in the group at 2.5 individuals/L [13]. Tian et al. also reported that the activities of ACP and AKP in *Scylla paramamosain* cultivated under high stocking density were significantly lower than those under low density [30]. Collectively, it can be inferred that elevated stocking density inhibits the activities of ACP and AKP, thereby impairing the immune function of *P. monodon*.

Elevated stocking density is a recognized environmental stressor that disrupts the balance between reactive oxygen species (ROS) production and elimination, often overwhelming the endogenous antioxidant defense system [31]. In the present study, the significant increase in MDA content with rising density provides direct evidence of lipid peroxidation and cellular oxidative damage, a consequence consistent with findings in *Ictalurus punctatus* where high density led to increased MDA levels [32]. Meanwhile, the obvious decrease in important antioxidant components (T-AOC, SOD activity, GSH content) indicates an impairment of antioxidant capacity, a common response to chronic stress observed in *Oncorhynchus mykiss* and *Salminus brasiliensis* [33,34]. The impairment of these antioxidant defenses reduces the ability to clear excess ROS, leading to increased oxidative damage. This oxidative damage is directly linked to the reduced growth, suggesting a direct connection between the stress caused by high density and poorer shrimp performance. Remarkably, our results indicated a distinct increase in CAT activity under high density, which may represent a compensatory mechanism. In contrast to the downregulation of SOD and the glutathione system, the increase in CAT activity likely represents an adaptive response to scavenge hydrogen peroxide and mitigate hydroxyl radical formation. Similar compensatory induction of CAT activity upon high-density stress was found in *Pelteobagrus fulvidraco*, where elevated serum CAT levels corresponded to a decline in GSH [28]. Together, these findings underscore that the antioxidant system of *P. monodon* responds in a non-uniform manner under high stocking density, with certain pathways being upregulated to partially counterbalance the impairment of others.

With the advance of sequencing technology, RNA-seq has become a widely used approach in crustaceans for identifying genes and pathways involved in environmental responses [18,19,20]. In the present study, a total of 2284 genes were differentially expressed, suggesting their involvement in the high stocking density treatment. Subsequently, WGCNA was applied to move beyond individual gene lists and discover coordinated transcriptional programs. By grouping genes with similar expression patterns, this method uncovers modules of co-expressed genes which are often functionally related. These modules may be associated with the regulation of specific biological traits. To date, the WGCNA method has been extensively validated in studies of various aquatic species, confirming its reliability for extracting biologically meaningful networks from transcriptomic data [35,36]. Enrichment analysis of DEGs within the key modules (green and turquoise) provided an effective approach to study the molecular mechanisms of *P. monodon* in response to different stocking densities.

The high stocking density dramatically altered the metabolic profile of *P. monodon*, especially in core metabolic pathways. The green module, associated with the medium density (G2), was enriched for processes related to amino acid (e.g., tyrosine, arginine and proline, and alanine/aspartate/glutamate metabolism) and lipid (e.g., glycerophospholipid and sphingolipid metabolism, biosynthesis of unsaturated fatty acids) metabolism. This indicates that shrimp in moderately stocked conditions can maintain a balanced allocation of resources, thereby supporting physiological homeostasis and promoting long-term health. The same trend was observed in our previous work, where moderate stress maintained a balance between cellular homeostasis and reproductive functions [37]. In contrast, the turquoise module associated with high density (G3) showed significant enrichment for oxidoreductase activity and detoxification (xenobiotics/drugs via cytochrome P450 enzymes), indicating a reconfiguration of cellular machinery for handling stress. This stress-responsive signature was also reported in *Portunus trituberculatus* and *Macrobrachium nipponense*, which highlighted the enrichment of oxidoreductase and detoxification processes in response to environmental stress [38,39]. Notably, lysine degradation and pyruvate metabolism were also enriched in the turquoise module, which could provide energy for cells via the TCA cycle [40,41]. This transition from general homeostasis to specific stress response implies that under high-density conditions, energy and resources are diverted away from long-term functions like reproduction and toward immediate survival. The high energy demand of the stress response is likely a key factor contributing to the shrimp’s decreased growth, which aligns with the trends in our growth performance metrics.

Increased stocking density is reported to reduce disease resistance and increase susceptibility to pathogens in aquatic animals [5]. In *L. vannamei*, high density accelerates white spot disease progression, resulting in earlier mortality peaks [42]. Similarly, the enrichment of immune and disease-associated pathways in our study suggests that high stocking density potentially elevates the risk of infection and physiological dysregulation in *P. monodon*. GO results showed that shrimp exposed to medium density (G2) activated basic immune processes, including humoral and innate immune responses. A similar reaction was found in *Ctenopharyngodon idellus*, where stocking density significantly altered the expression of immune-related genes [43]. This indicates that *P. monodon* initiates a proactive immune adaptation at medium densities to mitigate environmental pressure. In contrast, shrimp at high density (G3) exhibited a transcriptomic signature shift toward stress and disease states, with significant enrichment in pathways such as legionellosis, lipid-mediated inflammation, and chemical carcinogenesis. This enrichment implies that high stocking density likely dysregulates immune function and increases pathogen susceptibility [44]. The molecular-level disturbance further compromises physiological homeostasis. In our study, the significantly reduced activities of immune-related enzymes (ACP and AKP) align with this interpretation. Numerous studies across aquatic species have confirmed that excessively high stocking density suppresses immune enzyme activity, compromises tissue integrity, and elevates disease risk [45,46,47]. In summary, our integrated transcriptomic and physiological results elucidate the molecular mechanisms by which high density stress increases disease infection risk in *P. monodon*. It triggers a shift from effective immune regulation to chronic inflammatory and pathological states, which ultimately damages tissues and suppresses immune function.

## 4. Materials and Methods

### 4.1. Shrimp and Experimental Management

The shrimps used for experimentation were obtained from the Shenzhen experimental base of the South China Sea Fisheries Research Institute, Chinese Academy of Fisheries Sciences, Guangzhou, China. For experimental treatment, shrimps (4.5 ± 0.5 g in body weight) were randomly divided into three groups at stocking densities of 50 (G1), 100 (G2), and 150 (G3) individuals per cubic meter (ind/m^3^). Three plastic tanks (containing 0.5 m^3^ seawater per tank) were allocated to each experimental group. The experiment was conducted for 40 days while maintaining the water conditions at 28–30 °C in temperature, 30–32 in salinity, >10 mg/L in dissolved oxygen, 7.8–8.2 in pH, and <1.0 mg/L in ammonia. Shrimps were fed with commercial feed (Dongteng Feed Co., Ltd., Zhanjiang, China) 3 times a day, at 6:00, 14:00 and 22:00. Approximately half of the water in each tank was replaced daily.

### 4.2. Growth and Survival Assessment

Following the 40-day culture period, total harvest was conducted across all tanks. Each shrimp was individually weighed, and surviving specimens were enumerated to calculate survival rate. Growth and survival performance were evaluated using the following indices:

Weight gain rate (WGR, %) = [(Final weight − Initial weight)/Initial weight] × 100

Specific growth rate (SGR, % day^−1^) = [(ln Final weight − ln Initial weight)/Days] × 100

Feed conversion ratio = Feed intake/(Final body weight − Initial body weight)

Survival rate (SR, %) = (Final shrimp count/Initial shrimp count) × 100

### 4.3. Physiological and Biochemical Index Detection

To assess the effects of stocking density, immunity and antioxidant activities were measured at the beginning (0 d) and the end (40 d) of the experiment. The hepatopancreas tissue was selected for physiological and biochemical index detection. According to the requirements of the instruction manual (Nanjing Jiancheng Biotechnology Co., Ltd., Shanghai, China), sample pretreatment, reagent preparation and sample detection were conducted. In this research, the enzymatic activities of AKP, ACP, T-AOC, SOD and CAT were tested following the kits encoded as A059-1-1, A060-1-1, A015-2-1, A001-1-2 and A007-1-1. In addition, the contents of MDA and GSH were measured using the kits encoded as A003-1-2 and A006-1-1, respectively.

### 4.4. Transcriptome Sequencing and Analysis

Approximately 100 mg of hepatopancreas tissue was harvested, thoroughly homogenized in liquid nitrogen, and then transferred to a pre-chilled 1.5 mL centrifuge tube containing 1 mL of Trizol reagent. Total RNA was isolated using the MJZol Total RNA Extraction Kit (Shanghai Majorbio Bio-pharm Biotechnology Co., Ltd., Shanghai, China), strictly following the manufacturer’s protocols. Subsequently, the quality and quantity of the isolated RNA were assessed with an Agilent 5300 BioAnalyzer (Agilent Technologies, Santa Clara, CA, USA) and a NanoDrop 2000 spectrophotometer (Thermo Scientific, Waltham, MA, USA). Only high-integrity RNA samples meeting the criteria of OD260/280 ratio ranging from 1.8 to 2.2 and RNA Integrity Number (RIN) > 6.5 were selected for sequencing library construction.

Transcriptome libraries for *P*. *monodon* RNA-seq were constructed in accordance with the protocols of Illumina^®^ Stranded mRNA Prep, Ligation (San Diego, CA, USA), using 1 μg of total RNA. Subsequently, following quantification via a Qubit 4.0 fluorometer, the sequencing library was subjected to paired-end sequencing (PE150) on the DNBSEQ-T7 platform. Raw paired-end reads were processed for trimming and quality control using the fastp tool [48] with default parameters. After filtering, the clean reads were then individually mapped to the reference genome (GenBank accession number: GCF_015228065.2) using HISAT2 v2.2.1 [49] software. Mapped reads from each sample were subjected to reference-based assembly utilizing StringTie v2.2.3 [50].

In this study, expression levels of transcript were estimated using the transcripts per million reads (TPM) quantification approach. The quantification of gene abundance was performed by RSEM v1.3.3 [51]. Differential expression analysis was primarily conducted with the DESeq2 package v1.50.2 [52]. Genes with a false discovery rate (FDR)-adjusted *p*-value < 0.05 and an absolute log_2_ fold change (|log_2_FC|) ≥ 1 were screened as DEGs. WGCNA was conducted to identify functional modules related to different stocking densities [53]. To annotate the biological functions of the identified DEGs, functional enrichment analyses (including GO term and KEGG pathway analysis) were performed. Terms and pathways were considered significantly enriched if they met the condition of a Bonferroni-corrected *p*-value < 0.05 relative to the whole-transcriptome background.

### 4.5. Validation of DEGs Using Quantitative Real-Time PCR (qRT-PCR)

Ten target genes were randomly chosen for qRT-PCR to validate the reliability of the sequencing data. Primers were designed using Primer Premier v5.0 software, and EF-1α was selected as the internal control. Total RNA was extracted from hepatopancreas tissue using the TRIzol kit (Invitrogen, Carlsbad, CA, USA), followed by cDNA synthesis with the Evo M-MLV Reverse Transcription Kit (Accurate Biotechnology Co., Ltd., Guangzhou, China). Gene expression analysis was performed using a Roche Light Cycler^®^ 480II Real-Time PCR System (Roche, Basel, Switzerland). Three technical replicates were established for each sample and the internal reference gene.

### 4.6. Statistical Analysis

Statistical analyses of growth performance, physiological and biochemical index, and qRT-PCR results were performed using SPSS statistics version 23.0 software (IBM, Armonk, NY, USA). First, the data were tested for homogeneity of variances (*F* test). Then, a one-way analysis of variance (ANOVA) was conducted, followed by Tukey’s multiple range test. Statistical significance was set at *p* < 0.05.

## 5. Conclusions

This study systematically explored the effects of stocking density on *P*. *monodon* at physiological and molecular levels. Key findings indicated the shrimp cultured at medium density (100 ind/m^3^) maintained normal physiological homeostasis. In contrast, high density (150 ind/m^3^) impaired growth performance and survival of shrimp, accompanied by suppressed non-specific immune function, disrupted antioxidant balance and altered metabolic patterns. These results clarify the molecular mechanisms of *P. monodon*’s response to different stocking densities and provide practical guidance for optimizing stocking density in industrial aquaculture.

## Figures and Tables

**Table 1:** Summary of transcriptome data.

Sample	Raw Reads	Clean Reads	Error Rate (%)	Q20 (%)	Q30 (%)	GC Content (%)
G1_1	61,898,742	61,896,516	0.0196	99.41	97.97	52.91
G1_2	45,663,312	45,661,780	0.0205	99.22	97.42	49.43
G1_3	50,345,378	50,344,086	0.0201	99.32	97.66	53.75
G2_1	46,989,524	46,987,290	0.0200	99.30	97.69	50.29
G2_2	42,619,338	42,618,228	0.0202	99.30	97.61	53.64
G2_3	61,304,616	61,301,208	0.0199	99.37	97.83	52.83
G3_1	40,891,838	40,890,994	0.0201	99.34	97.72	49.56
G3_2	49,214,590	49,213,434	0.0202	99.25	97.57	47.51
G3_3	43,481,080	43,479,496	0.0197	99.40	97.94	47.12

## Data Availability

The mRNA sequencing data generated in this study have been deposited in the Genome Sequence Archive at the National Genomics Data Center, China National Center for Bioinformation, under accession number GSA: CRA037498 (accessible via https://ngdc.cncb.ac.cn/gsa/browse/CRA037498; accessed on 29 January 2025).
